# Antagonizing αvβ3 Integrin Improves Ischemia-Mediated Vascular Normalization and Blood Perfusion by Altering Macrophages

**DOI:** 10.3389/fphar.2021.585778

**Published:** 2021-02-24

**Authors:** Yongjie Li, Qian Gao, Xin Shu, Lamei Xiao, Yan Yang, Ningbo Pang, Yulin Luo, Jing He, Liping Zhang, Jianbo Wu

**Affiliations:** ^1^ Key Laboratory of Medical Electrophysiology of Ministry of Education, Collaborative Innovation Center for Prevention and Treatment of Cardiovascular Disease of Sichuan Province, Drug Discovery Research Center, Southwest Medical University, Luzhou, China; ^2^ Laboratory for Cardiovascular Pharmacology, Department of Pharmacology, School of Pharmacy, Southwest Medical University, Luzhou, China

**Keywords:** integrin, cilengitide, angiogenesis, macrophage, ischemia, vascular

## Abstract

**Background**: αVβ3 integrin has been implicated in the physiological processes and pathophysiology of important angiogenesis-related disorders; however, the preclinical and clinical data on integrin αVβ3 antagonists have not demonstrated improved outcomes. Our goal was to test the hypothesis that inhibition of αVβ3 integrin improves blood flow in a mouse hindlimb ischemia model.

**Methods**: In this study, we examined the effect of cilengitide, an αVβ3/αVβ5 integrin-specific RGD-mimetic cyclic peptide, on blood perfusion and angiogenesis after hindlimb ischemia. Blood flow was measured using Laser Doppler Scanner. Vascular density, and macrophages infiltration were examined by immunofluorescence. Macrophage polarization was measured by quantitative real time PCR.

**Results**: We found that low-dose, not high-dose, cilengitide increased blood flow perfusion, capillary formation, and pericyte coverage, accompanied by an accumulation of macrophages and increased expression of the chemokine (C-C motif) ligand 2 (CCL2) in ischemic muscles. Macrophage depletion using clodronate liposomes resulted in a reduction in low-dose cilengitide-induced blood flow perfusion, macrophage accumulation, pericyte coverage, and CCL2 expression. Finally, *in vitro* assays showed that low-dose, not high-dose, cilengitide increased macrophage migration.

**Conclusion**: These studies identified a novel role of the inhibition of αVβ3 integrin in modulating ischemia-induced angiogenesis, possibly through effects on macrophage infiltration and polarization, and revealed αVβ3 integrin inhibition to be a promising therapeutic strategy for peripheral artery disease.

## Introduction

Integrins are heterodimeric cell surface receptors that directly bind components of the extracellular matrix (ECM) and participate in angiogenic endothelial cell functions (Brooks et al., 1994; Desgrosellier and Cheresh, 2010; Kim et al., 2011; Weilbaecher et al., 2011). Integrins are highly expressed in vessels only when angiogenesis occurs, but few are expressed in mature vessels (Luo et al., 2007). Therefore, inhibitors targeting αVβ3 and αVβ5 integrins have been developed to treat diseases involving abnormal angiogenesis such as tumors (Katsamakas et al., 2017). Cilengitide, an inhibitor of αVβ3 and αVβ5 integrins, exerts both proangiogenic and antiangiogenic properties, and the concentration of cilengitide is an important determinant of which effect is observed. In both *in vitro* and animal models, a high dose of cilengitide was found to inhibit tumor cell proliferation, migration, and neoangiogenesis (Mas-Moruno et al., 2010). The proangiogenic effect of cilengitide seems to be mediated by promoting VEGF-mediated angiogenesis via altering α_V_β_3_ integrin and vascular endothelial growth factor receptor-2 trafficking (Reynolds et al., 2009). Unfortunately, cilengitide failed to improve survival benefit in clinical trials for many cancer types when administered at high doses (5 mg/kg) (Bridges and Harris, 2015; Wong et al., 2015).

αVβ3 integrin is minimally expressed in normal quiescent endothelial cells but significantly upregulated during neovascularization. A previous study showed CD31-positive endothelial cells within the capillaries and small arterioles of non-diabetic ischemic hindlimb sections, which correlated with high αVβ3 expression (Hedhli et al., 2018). However, this finding has not been tested in a hindlimb ischemic disease model.

In this study, we show for the first time, to our knowledge, that treatment with a low dose of cilengitide normalizes vasculature, increases macrophage infiltration, and promotes blood perfusion in ischemic muscles in a mouse hindlimb ischemia model. By enhancing vasculature maturation and increasing macrophage infiltration within ischemic muscles, our data provide a promising therapeutic strategy for peripheral artery disease. Furthermore, since cilengitide, an integrin αVβ3 antagonist, has been approved for chemotherapeutics, our findings could be rapidly tested in the clinic to enhance peripheral artery disease treatment.

## Material and Methods

### Reagents

Clodronate liposomes were purchased from Roche and prepared as described previously (Van Rooijen et al., 1994). Clodronate liposomes have been used extensively to specifically deplete macrophages *in vivo* by inducing apoptosis in these cells without affecting other cell types. Cilengitide was obtained from Med Chem Express (NJ, United States).

### Animals

C57BL/6J mice were obtained from the Chongqing Medical University Animal Center, Chongqing, China. All animal care and experimental procedures were approved by the Southwest Medical University Animal Care and Use Committee.

### Animal Model of Type 2 Diabetes

Six-week-old male C57BL/6J mice were fed a high fat diet (HFD) (TP2330055A; Research Diet, Trophic Animal Feed High-tech Co. Ltd, China**)** for 14 weeks. After the HFD treatment for 14 weeks, mice were intraperitoneally injected with streptozocin (STZ) (85 mg/kg/d). One week later, the blood glucose higher than 11.1 mM were randomly divided into three groups (n = 6). Blood samples were obtained from the tail vein and blood glucose levels were measured using an automatic glucometer (Accu-Chek; Roche Diagnostics, Mannheim, Germany).

### Mouse Hindlimb Ischemia Model

Unilateral hindlimb ischemia was induced in mice by ligation and excision of a segment of the left femoral artery, as previously described (Xiao et al., 2016). Mice were anesthetized with sodium pentobarbital (60 mg/kg body weight, intraperitoneally)or isoflurane (5%, by inhalation) and a single subcutaneous dose of buprenorphine hydrochloride (0.1 mg/kg) for analgesia. Additional sodium pentobarbital (12 mg/kg body weight) or 5% isoflurane was given as needed to maintain anesthesia. The depth of anesthesia was evaluated by pinching to stimulate the pedal withdrawal reflex. Mice were euthanized by cervical dislocation at the end of the experiment while still anesthetized. For cilengitide treatment, mice were administered cilengitide 50 μg/kg or 5 mg/kg by intraperitoneal injection twice a week after undergoing unilateral right femoral artery ligation surgery. For the clodronate liposome treatment, the treatment was administered intravenously (100 μL liposomes, 0.5% clodronate) every 4 days in the presence or absence of a low dose of cilengitide. Perfusion of the ischemic and nonischemic hindlimb was measured in each mouse by laser-Doppler imaging (LDI) immediately before surgery, 1 h after surgery, and at several subsequent time points using a moorLDI2-HIR high-resolution laser Doppler imager (Moor Instruments). Mice were euthanized 14 days after surgery.

### Evaluation of Hindlimb Function and Ischemic Damage

Modification of a clinical standard score for hindlimb ischemia was used serially for semiquantitative assessment of hindlimb function (0: normal, 1: plantar flexion and grasping, 2: plantar flexion, and 3: dragging of foot) (Tilan et al., 2013).

### Immunofluorescence

Middle sections of the isolated ischemic gastrocnemius muscle were fixed in 4% paraformaldehyde, embedded in paraffin, and serially cut into 10-mm-thick sections. The sections were incubated with anti-F4/80, anti-PECAM-1, and anti-smooth muscle *α*-actin antibodies (Santa Cruz Biotechnology Inc, Santa Cruz, CA). The sections were incubated with goat anti-rabbit IgG AlexaFluor 488-conjugated antibody or goat anti-mouse IgG AlexaFluor 568-conjugated antibody (both Molecular Probes, Invitrogen). Images were captured using an Olympus (DP70) microscope (Melville, NY) and evaluated using the Photoshop CS4 (Adobe, San Jose, CA) function. Capillaries within the gastrocnemius muscle were immunostained with anti-PECAM-1 antibody, and the percentage of the area positive for PECAM-1 or total number of capillaries around each fiber was determined throughout entire cross-sectional regions. Quantification was performed by analyzing at least three sections and three fields per sample.

### Quantitative Real-Time PCR

Total RNA was extracted from hindlimb muscles using TRIzol (Invitrogen). RNA samples were pretreated with deoxyribonuclease I (Invitrogen Life Technologies) and cDNA synthesized using a SuperScript kit (Invitrogen Life Technologies). Quantitative RT-PCR was performed with the ABI PRISM 7700 System (Applied Biosystems, Foster City, CA) according to the manufacturer’s recommendations. Each sample was analyzed in duplicate with ribosomal 18S RNA used as an internal control. The quantitative real-time (qRT-PCR) products were also validated by ethidium bromidestained 2.0% agarose gel electrophoresis after end-point PCR. Fold changes in gene expression were determined using the 2−ΔΔCT method. The results are presented as the mean ± SEM. Sequences of all primers are shown in Supporting Table 1.

**TABLE 1:** Sequences of primers used in the study.

CD11c	Forward	CCT​CAA​GAC​AGG​ACA​TCG​CT
NM_021334.2	Reverse	CAC​TCA​GTG​ACT​GCC​CAA​AA
IL-1β	Forward	ACT​ACA​GGC​TCC​GAG​ATG​AAC​AAC
NM_008361.4	Reverse	CCC​AAG​GCC​ACA​GGT​ATT​TT
CCL2	Forward	ATGCAGGTCCCTGTCATG
NM_011333.3	Reverse	GTT​CAC​TGT​CAC​ACT​GGT​CA
IL-10	Forward	TGT​CAA​ATT​CAT​TCA​TGG​CCT
NM_010548.2	Reverse	ATC​GAT​TTC​TCC​CCT​GTG​AA
TGFβ1	Forward	TGC​TAA​TGG​TGG​ACC​GCA​A
NM_011577.2	Reverse	CAC​TGC​TTC​CCG​AAT​GTC​TGA
CD206	Forward	CAT​GGA​TGT​TGA​TGG​CTA​CTG​GAG
NM_008625.2	Reverse	GTC​TGT​TCT​GAC​TCT​GGA​CAC​TTG
18S rRNA	Forward	GCA​ATT​ATT​CCC​CAT​GAA​CG
NR_003278.3	Reverse	GGC​CTC​ACT​AAA​CCA​TCC​AA

### ELISA

Lysates from hind limb muscle tissue homogenates harvested at day 14 post-surgery were used for ELISA analysis. VEGF-A was measured using a mouse VEGF-A total antigen assay ELISA kit (R and D systems). The values were normalized to total protein levels assessed with a bicinchoninic acid (BCA) protein assay (Pierce). The ratio of protein in the ischemic to the nonischemic muscle was calculated for each mouse.

### Cell Culture

RAW 264.7 cells (ATCC) were seeded in 6-well plates (0.5×10^6^ cells/cm^2^) and grown in RPMI 1640 supplemented with 10% FBS and antibiotics. Adherent RAW 264.7 cells were trypsinized and counted, and then they were added to different concentrations of cilengitide in serum-free medium for 24 h.

### Isolation of Peritoneal Macrophages

Normal diet 57BL/6J mice were intraperitoneally injected with 1 ml of a 3% sterile thioglycollate solution (Difco Laboratories, Sparks, MD). After 4 days, peritoneal cells were harvested by washing the peritoneal cavity with PBS. Collected elicited-macrophages from peritoneum were counted by microscopy.

### Transwell Migration Analysis

A transwell migration assay was performed using 24-well transwell inserts (8-μm pore size, Corning). RAW 264.7 cells suspended in 150 μL of serum-free medium (2 × 10^5^ cells/mL) were incubated with a range of concentrations of cilengitide or PBS were seeded on the basal side of the insert for 24 h at 37°C in 5% CO2 (Su X et al., 2016). Then, the cells were fixed with 4% paraformaldehyde for 10 min at room temperature for subsequent crystal violet staining. The number of RAW 264.7 cells that migrated through the micropores was then counted at ×200 magnification with a light microscope. Each trial consisted of three independent samples, and all the assays were repeated two to three times.

### Statistical Analysis

Statistical analyses were performed using SPSS 16.0 software. Data are presented as the means ± SEM. Experimental groups were compared by 1-way ANOVA with a Dunnett multiple comparison test. Differences were considered significant at a *p* value of <0.05.

## Results

Low doses of cilengitide promote blood flow recovery and angiogenesis in a mouse hindlimb ischemia model.

**FIGURE 1:**
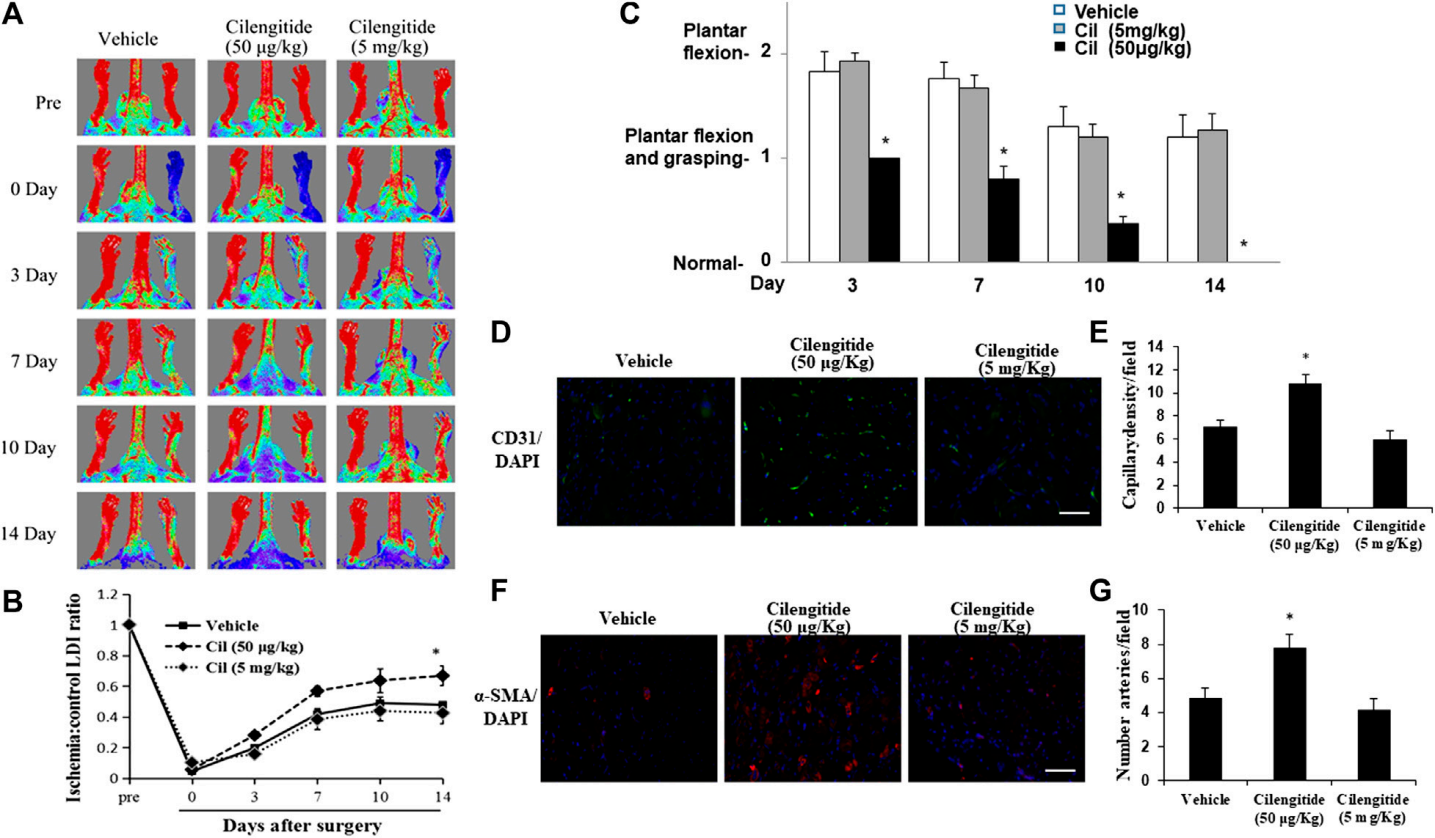
A low dose of cilengitide promoted limb ischemia blood flow recovery and angiogenesis. **(A)** Representative LDI images (n = 6 per group) of vehicle, cilengitide (50 μg/kg) and cilengitide (5 mg/kg) from Doppler monitoring before surgery, immediately after surgery or 14 days after surgery. **(B)** Quantitative analysis of the ratio of LDI in ischemic limbs compared to the nonischemic limbs of the same mice in the vehicle, cilengitide (50 μg/kg) and cilengitide (5 mg/kg) groups at the indicated time points after surgery. The results are shown as the mean ± SEM (n = 6). *p < 0.05 vs. vehicle and cilengitide (5 mg/kg) **(C)** Hindlimb ischemia clinical score: 0, normal; 1, plantar flexion and grasping; 2, plantar flexion; 3, dragging. A higher score indicates impaired functions. Data are presented as means ± SEM. *p < 0.05 vs. vehicle and cilengitide (5 mg/kg). **(D)** Immunostaining of the gastrocnemius capillary with a monoclonal antibody against PECAM-1 (green in tissue sections) in ischemic muscles in vehicle, cilengitide (50 μg/kg) and cilengitide (5 mg/kg) mice. **(E)** Quantitative analysis of capillary density at day 14 after surgery. Capillary density is expressed as the number of capillaries per high-power field (×200). Scale bars, 100 μm. The results are shown as the mean ± SEM (n = 6 per group). *p < 0.05 vs. vehicle and cilengitide (5 mg/kg). **(F)** Staining with *α*-smooth muscle actin (α-SMA; red) suggested a significantly increased number of arterioles in ischemic muscles from cilengitide (50 μg/kg)-treated mice compared with those from vehicle- and cilengitide (5 mg/kg)-treated mice at 14 days post ligation. **(G)** Quantitative analysis of arteriole density at day 14 after surgery. Arteriole density is expressed as the number of arterioles per high-power field (×200). Scale bars, 100 μm. The results are shown as the mean ± SEM (n = 6 per group). *p < 0.05 vs. vehicle and cilengitide (5 mg/kg).

A previous study indicated that low doses of cilengitide (50 μg/kg) stimulated tumor angiogenesis and promoted normalization of tumor vasculature (Wong et al., 2015). C57BL/6J mice received vehicle, cilengitide 50 μg/kg or 5 mg/kg by intraperitoneal injection twice a week after undergoing unilateral right femoral artery ligation surgery. Hindlimb blood flow was measured by real-time laser Doppler imaging (LDI) before surgery and on postoperative days 0, 3, 7, 10 and 14. LDI revealed that recovery of blood perfusion in the ischemic hindlimb after femoral artery interruption was significantly increased in cilengitide 50 μg/kg-treated mice compared to that in vehicle-and cilengitide 5 mg/kg-treated mice (Figures 1A,B). Importantly, there was no significant difference in blood perfusion between vehicle- and cilengitide 5 mg/kg-treated mice. Mice were further given a clinical score from 0 to 1 at each time point. Cilengitide 50 μg/kg-treated mice exhibited remarkable recovery on a score from one to 0 14 days after femoral artery ligation. In contrast, vehicle-and cilengitide 5 mg/kg-treated mice failed to display full use of hindlimb function by day 14. To further examine significance of cilengitide in a pathological model that is relevant to human cardiovascular disease, we fed mice high-fat diet (HFD) for 14 weeks, which produced obesity and hyperglycemia. After 14 days of treatment, Laser Doppler imaging revealed that recovery of perfusion in ischemic hindlimb tissue after femoral artery interruption was significantly increased in cilengitide 50 μg/kg-treated HFD mice compared to vehicle-and cilengitide 5 mg/kg-treated HFD mice (Supplementary Figure 1A-B). In contrast to normal diet mice, the basic level of blood flow perfusion in vehicle-HFD mice was significantly impaired (Figures 1A,B). These results suggest that a low concentration of cilengitide enhances mouse hindlimb ischemia blood flow recovery compared with a high concentration.

Consistent with these results, the density PECAM-1-positive capillaries (Figures 1D,E) and *α*-SMA-positive arterioles (Figures 1F,G) in ischemic gastrocnemius muscles 14 days after induction of ischemia was significantly increased in cilengitide 50 μg/kg-treated mice compared with that in vehicle- and cilengitide 50 mg/kg-treated mice.

### Low-Dose Cilengitide Treatment Promotes VEGF Expression in Ischemic Muscles

**FIGURE 2:**
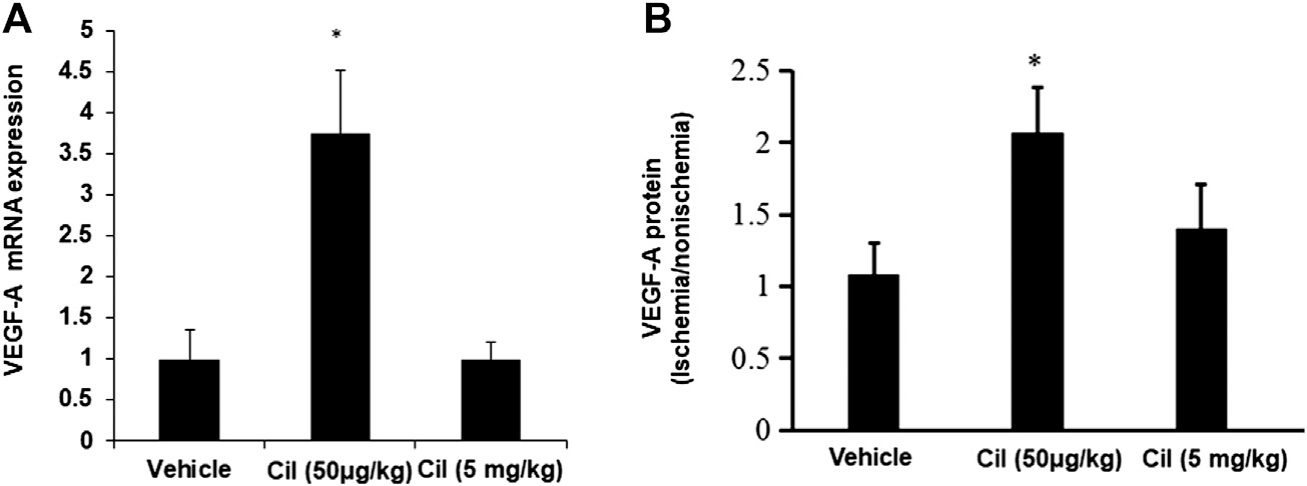
**A low dose of cilengitide increased VEGF-A expression in ischemic muscles. (A)**The levels of VEGF-A mRNA expression were measured by quantitative RT-PCR in nonischemic and ischemic muscle at 14 days after surgery. The results are shown as the mean ± SEM (n = 6 per group). *p < 0.05 vs. vehicle and cilengitide (5 mg/kg). **(B)** VEGF-A expression levels detected by ELISA in gastrocnemius muscles from 14 days after surgery. The ratio of protein in the ischemic to the nonischemic muscle was calculated for each mouse. The results are shown as the mean ± SEM (n = 6 per group). *p < 0.05 vs. vehicle and cilengitide (5 mg/kg).

A previous study demonstrated that treatment with low concentrations of the αVβ3/αVβ5 inhibitor mediated VEGF signaling (Reynolds et al., 2009). Thus, we next tested the direct effects of cilengitide on VEGF expression in ischemic muscles. Quantitative analysis by real-time polymerase chain reaction revealed that at 14 days after surgery, mice treated with cilengitide 50 μg/kg showed upregulated VEGF-A gene expression in ischemic muscles compared with the vehicle- and cilengitide 5 mg/kg-treated mice (Figure 2A). Similar results were observed at the protein level (Figure 2B). These results suggested that a low dose of cilengitide treatment promotes VEGF expression in ischemic muscles.

### A Low Dose of Cilengitide Promotes Macrophage Infiltration and Polarization

**FIGURE 3:**
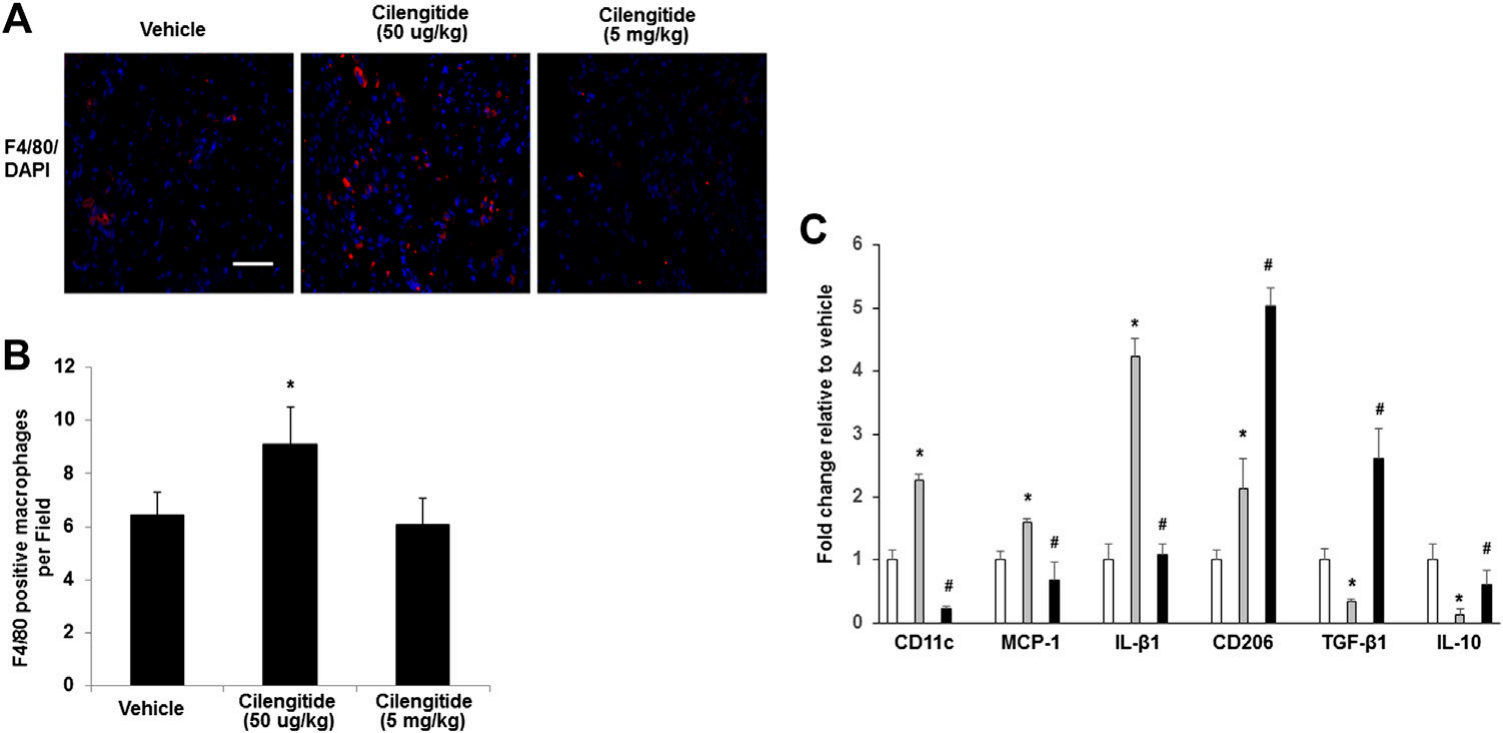
**Macrophages display an M1 phenotype in ischemic muscles in response to low doses of cilengitide. (A)** Representative images of macrophages as assessed by F4/80 immunostaining in ischemic gastrocnemius muscles in vehicle, cilengitide (50 μg/kg) and cilengitide (5 mg/kg) mice at 14 days after surgery. Scale bars, 100 μm. **(B)** Quantification of anti-F4/80 positive-macrophage infiltration of ischemic muscles. **(C)** The gene profile of the M1-and M2-macrophage phenotypes by quantitative RT-PCR of ischemic gastrocnemius muscles 14 days after surgery. All bars show the mean ± SEM (n = 6 per group). *p < 0.05 vs. vehicle; #*p* < 0.05 vs. cilengitide (50 μg/kg).

A previous study revealed that inhibition of αVβ3 integrin leads to efficient immune cell recruitment and macrophage polarization (Su et al., 2016). We therefore examined whether the treatment of cilengitide correlated with differences in macrophage infiltration or with their polarization state in ischemic muscles. Interestingly, we found that the cilengitide 50 μg/kg-treated mice showed a significant increase in the total macrophage number (F4/80-positive) in ischemic muscles compared with the vehicle- and cilengitide 5 mg/kg-treated mice after 14 days (Figures 3A,B). To further investigate the role of cilengitide in the polarization of the resident macrophages in ischemic muscles, transcript markers for the M1 and M2 macrophage phenotypes were evaluated by quantitative RT-PCR. Levels of proinflammatory M1 markers, including CD11c, CCL2, and IL-β1, in the ischemic muscles were significantly higher in the cilengitide 50 μg/kg-treated mice than in the vehicle- and cilengitide 5 mg/kg-treated mice (Figure 3C), although the levels of M1 marker in the high-dose cilengitide-treated mice were similar to those in the vehicle-treated mice. Moreover, we examined the anti-inflammatory M2 markers, including CD206, TGF-β1, and IL-10, in ischemic muscles and found that the levels of CD206, TGF-β1, and IL-10 were significantly lower in the cilengitide 50 μg/kg-treated mice than in the cilengitide 5 mg/kg-treated mice. However, only TGF-β1 and IL-10 levels were significantly decreased in the cilengitide 50 μg/kg-treated mice compared to those in the vehicle-treated mice (Figure 3C). Together, these results suggested that a low dose of cilengitide increases the infiltration of macrophages into the ischemic muscles and more specifically, promotes an M1 phenotype shift.

### Macrophage Depletion Attenuates the Low-Dose Cilengitide-induced Stimulation of Angiogenesis

**FIGURE 4:**
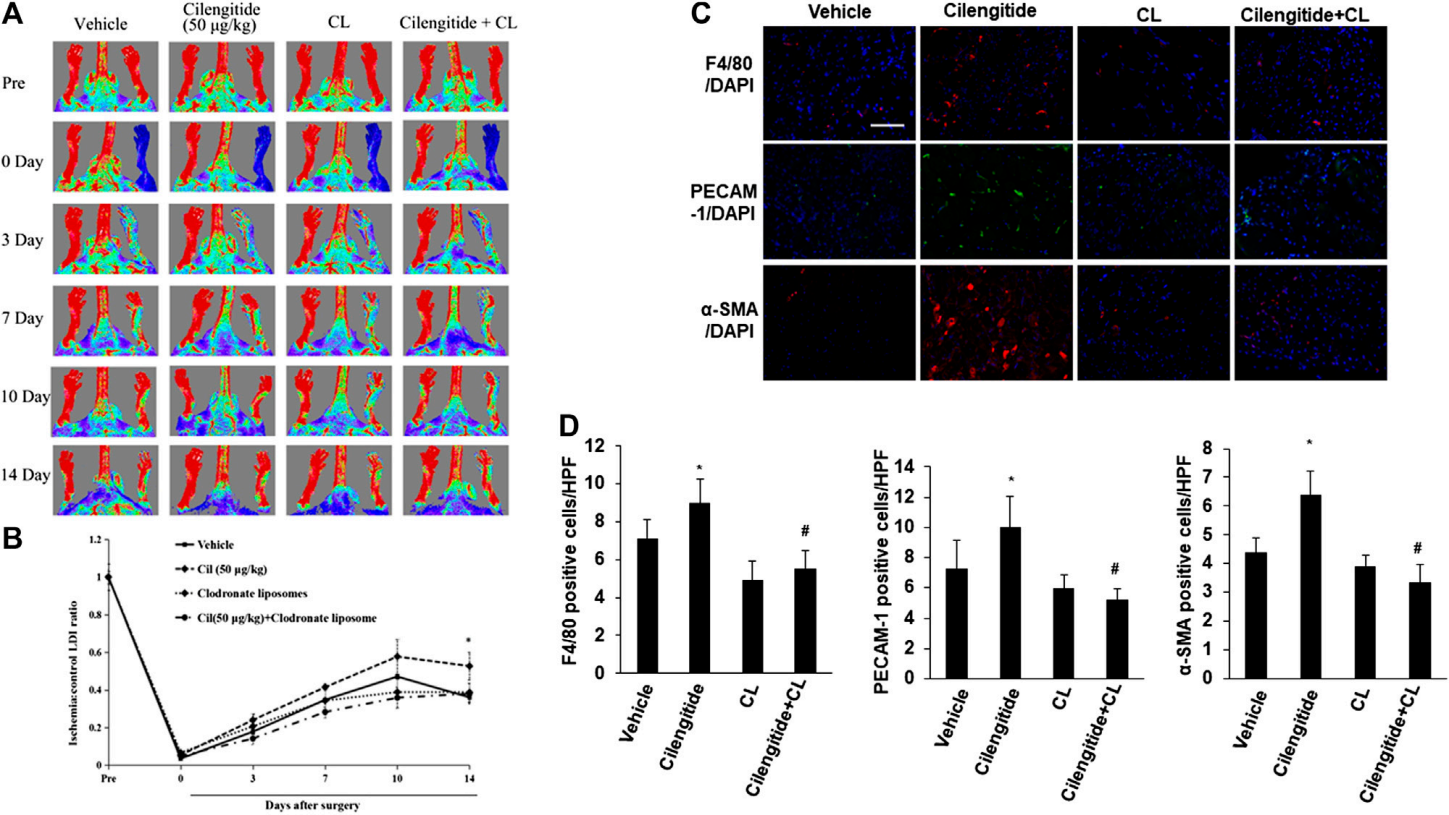
**Macrophages are involved in low-dose cilengitide-induced angiogenesis. (A)** Representative laser Doppler images of mouse hindlimbs at the indicated time points after femoral artery ligation. Pre and post indicate immediately before and after surgery, respectively. **(B)** Mean ratio of blood flow in ischemic and nonischemic hindlimb foot pads for all animals at the indicated time points (n = 6 per group; *p < 0.05 vs. vehicle control). **(C)** Representative images of F4/80, PECAM-1, and *α*-SMA immunostaining in ischemic gastrocnemius muscles recovered 14 days after femoral artery interruption. Scale bars, 100 μm **(D–F)** Mean F4/80-positive macrophages **(D)**, PECAM-1-positive capillary **(E)**, and *α*-SMA-positive arteriole density **(F)** in ischemic gastrocnemius muscle was assessed in vehicle, cilengitide (50 μg/kg), and cilengitide (50 μg/kg) + clodronate liposomes (100 μL liposomes, 0.5% clodronate), and clodronate liposomes alone group (n = 6 per group). *p < 0.05 vs. vehicle control; #*p* < 0.05 vs cilengitide (50 μg/kg). CL: clodronate liposomes.

We next examined whether systemic depletion of monocytes/macrophages may influence the positive effects of low-dose cilengitide on blood perfusion. Clodronate liposomes have been used extensively to specifically deplete macrophages with long-term effects *in vivo* (Fraser et al., 1995; Xiao et al., 2016). Thus, placebo or clodronate liposomes were administered intravenously (100 μL liposomes, 0.5% clodronate) every 4 days in the presence or absence of the low dose of cilengitide. After 14 days of treatment, LDI revealed that the recovery of perfusion in the ischemic hindlimb by the low dose of cilengitide was significantly abrogated in clodronate-treated mice compared with the placebo-treated controls (Figures 4A,B). Consistent with these results, the increases in macrophage infiltration and capillary and arteriole density observed following treatment with a low dose of cilengitide in ischemic gastrocnemius muscles were significantly reduced in the clodronate liposome–treated mice compared with those in the controls (Figures 4C,D).

### A Low Dose of Cilengitide Increases Macrophage Migration *in vitro*

**FIGURE 5:**
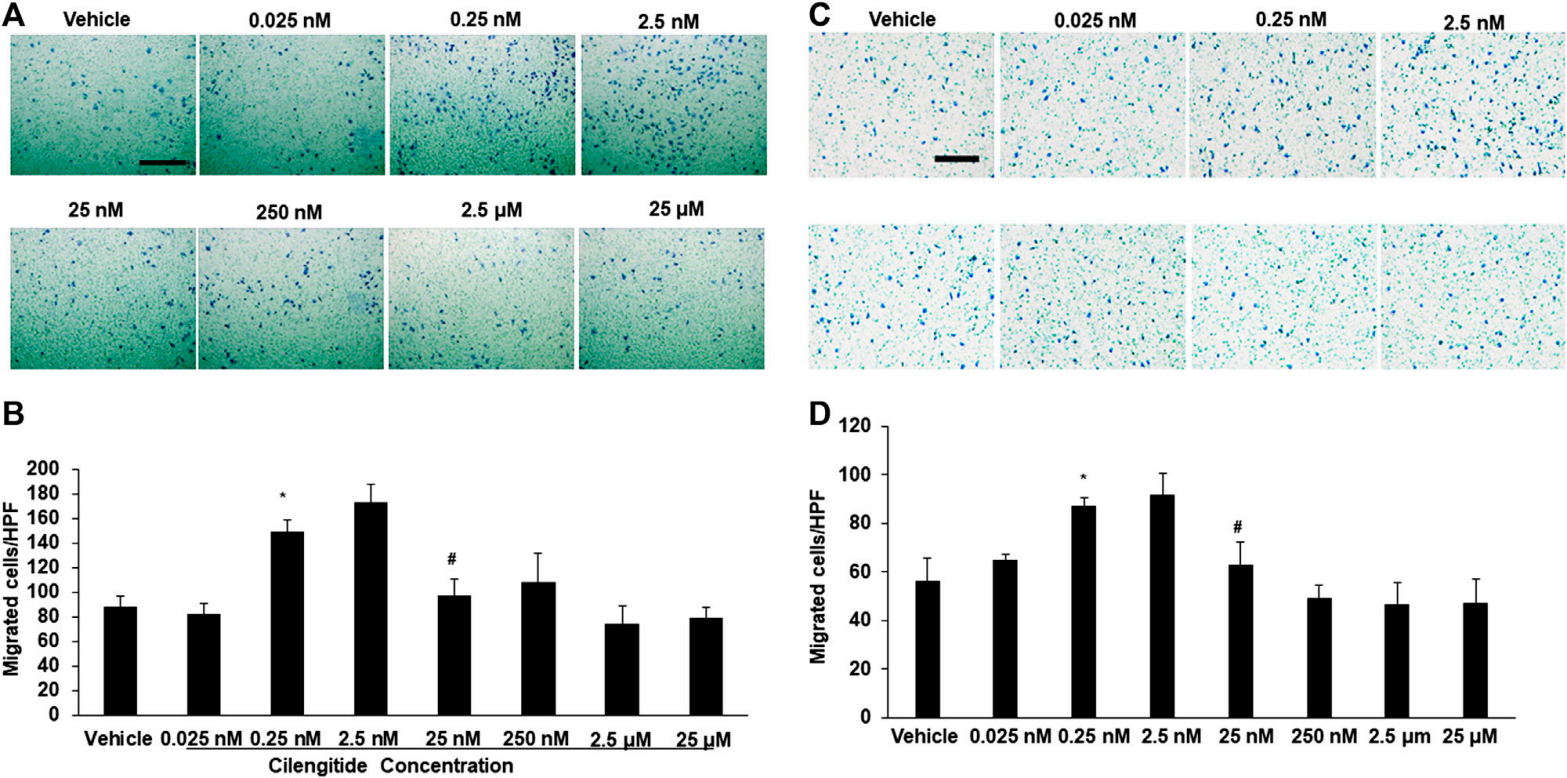
**A low dose of cilengitide promoted macrophage migration *in vitro*. (A)** Macrophage cell line RAW 24.7 migration measured after treatment with different doses of cilengitide for 24 h **(B)** Data are the means of triplicate experiments. *p < 0.05 vs. negative control (RAW 24.7 not exposed to cilengitide). #*p* < 0.05 vs. 0.25–2.5 nM cilengitide treatment. **(C)** The isolated C57/6J mice peritoneal macrophages were added to upper chambers. As indicated, different dose of cilengitide were put in the lower chambers. Cells were fixed after 24 h and stained with crystal violet. Migrated cells were counted. Representative images of cell migration are shown. **(D)** Quantitative assessment of triplicate cell migration experiments was performed. Data shown are mean ± SEM. *p < 0.05 Vs. vehicle. Scale bars, 100 μm. HPF indicates a high-power field.

We conducted *in vitro* experiments to examine the potential mechanisms underlying the effects of cilengitide *in vivo*. RAW 264.7 cells, a macrophage cell line, were incubated with a series of doses of cilengitide in transwell chambers. The results showed that after 24 h of treatment, a low dose of cilengitide (2.5 nM) significantly increased macrophage migration, though higher concentrations of cilengitide did not produce a significant effect (Figures 5A,B). We further examined the effect of cilengitide on macrophage migration using isolated C57BL/6J mice peritoneal macrophage. Similarly, the treatment of a low dose of cilengitide significantly promoted the migration of macrophages, but higher concentrations of cilengitide did not produce a significant effect (Figures 5C,D).

## Discussion

We described a previously unrecognized role of cilengitide in regulating ischemia-induced angiogenesis. Specifically, a low dose of cilengitide promoted the recovery of tissue perfusion, development of collateral arterioles after the induction of hindlimb ischemia, and increased macrophage infiltration to ischemic muscles, as well as macrophage polarization. Further, systemic blockade of macrophages by clodronate liposomes abrogated the recovery benefit of low-dose cilengitide treatment.

Similar to solid tumors, αVβ3 integrin is highly expressed in nondiabetic ischemic murine hindlimb (Hedhli et al., 2018). Our findings are consistent with previous reports indicating that a low dose of cilengitide promotes angiogenesis in tumor models (Reynolds et al., 2009; Wong et al., 2015). However, in contrast to tumor models, we observed additional benefits of VEGF expression in ischemic muscles. This result may reflect the greater dependence of ischemia-induced angiogenesis on VEGF, indicating that the effects of a low dose of cilengitide on vascularization were model- and tissue-specific.

A recent study demonstrated that inhibition of αVβ3 integrin leads to efficient immune cell recruitment and macrophage polarization (Su et al., 2016). Notably, depletion of integrin-β3 regulates the infiltration of macrophages and macrophage polarization and is more likely to polarize toward an M2 phenotype in different disease models (Fraser et al., 1995; Arras et al., 1998; Pollard, 2004; Qian and Pollard, 2010; Zhang et al., 2012). The application of a high dose of cilengitide was shown to enhance tumor growth (Wong et al., 2015; Su et al., 2016) and increase M2 function in some animal tumor models (Su et al., 2016). However, this study did not evaluate the effect of low-dose cilengitide on macrophage function. We observed that low doses of cilengitide, not high doses, increased macrophage infiltration in ischemic muscles *in vivo* and in a macrophage cell line *in vitro*, and more specifically, promoted the M1 phenotype shift in ischemic muscles. Additionally, treatment with the low dose of cilengitide inhibited the ischemia-promoting M2 phenotype, thus inducing proangiogenic properties. Furthermore, systemic blockade of macrophages by clodronate liposomes abrogated the blood perfusion recovery associated with low-dose cilengitide treatment in a hindlimb ischemia model. In other words, the single-agent clodronate effectively blocked the macrophage infiltration and angiogenesis induced by low-dose cilengitide in an established model.

Macrophage recruitment and both subtypes of macrophage polarization are required during remodeling of the vasculature, and the release of chemotactic factors, such as CCL2 and TNF-α by M1-macrophages enhanced recruitment of circulating leukocytes from the circulation (Troidl et al., 2013). Some studies, however, have demonstrated that M2 phenotype macrophages are purported to synthesize angiogenic cytokines such as VEGF-A and promote angiogenesis (Kodelja et al., 1997; Arnold et al., 2007). We observed that mice treated with a low dose of cilengitide had significantly increased expression of M1 markers in ischemic muscles compared with mice injected with saline. Consistent with these results, the number of *α*-SMA-positive smooth muscle cells (SMCs) (pericyte recruitment) in ischemic gastrocnemius muscle 14 days after induction of ischemia were significantly increased in low-dose cilengitide-treated mice compared with that in controls. Thus, increased outward remodeling into a mature capillary was observed in low-dose cilengitide-treated mice (Chalothorn et al., 2005). These findings indicate that treatment with a low dose of cilengitide is closely associated with vessel maturation through the recruitment of pericytes, suggesting that targeting αVβ3 integrin with a low dose of cilengitide plays an important role in the remodeling of the vasculature.

A limitation of our hindlimb ischemia model experiment is that we cannot definitively conclude that low-dose cilengitide promoted tissue blood perfusion solely by blocking the effect of αVβ3 integrin on macrophage activation because low-dose cilengitide could potentially modulate the angiogenic response to ischemia by inflammatory reaction-independent pathways (Pollard, 2004). Nevertheless, our hindlimb ischemic disease model data support the significance of our proposed improvements in ischemic perfusion in a clinically relevant *in vivo* context. To better understand what is being affected by cilengitide and when, it should be necessary to dissect further characterize the kinetics of vascular network formation and markers more carefully over time (4, 7, 14 days). Furthermore, we were limited by the measurement of the circulating inflammatory markers at a single time point.

Overall, our data suggest that targeting αVβ3 integrin with a low dose of cilengitide to promote ischemia-induced blood perfusion could provide an effective therapeutic approach in the clinic to improve peripheral artery disease treatment.

## Conclusion

Inhibition of αVβ3/αVβ5 integrins has failed to produce significant results in many cancer types. Cilengitide exerts proangiogenic properties at a low concentration in a murine tumor model as a potential driver of vasculature by blocking αVβ3 integrin in ischemic diseases. A low dose of cilengitide, not a high dose, is able to enhance blood perfusion in a murine hindlimb ischemia model. This effect suggests that a low dose of cilengitide has the potential to enhance treatment in patients with peripheral artery disease.

## Data Availability

The raw data supporting the conclusions of this article will be made available by the authors, without undue reservation, to any qualified researcher.
