# Supplementary figures and images for: Antagonizing αvβ3 Integrin Improves Ischemia-Mediated Vascular Normalization and Blood Perfusion by Altering Macrophages
Source: Front Pharmacol. 2021 Feb 24;12:585778. doi: 10.3389/fphar.2021.585778 (PMC7944575; doi:10.3389/fphar.2021.585778)

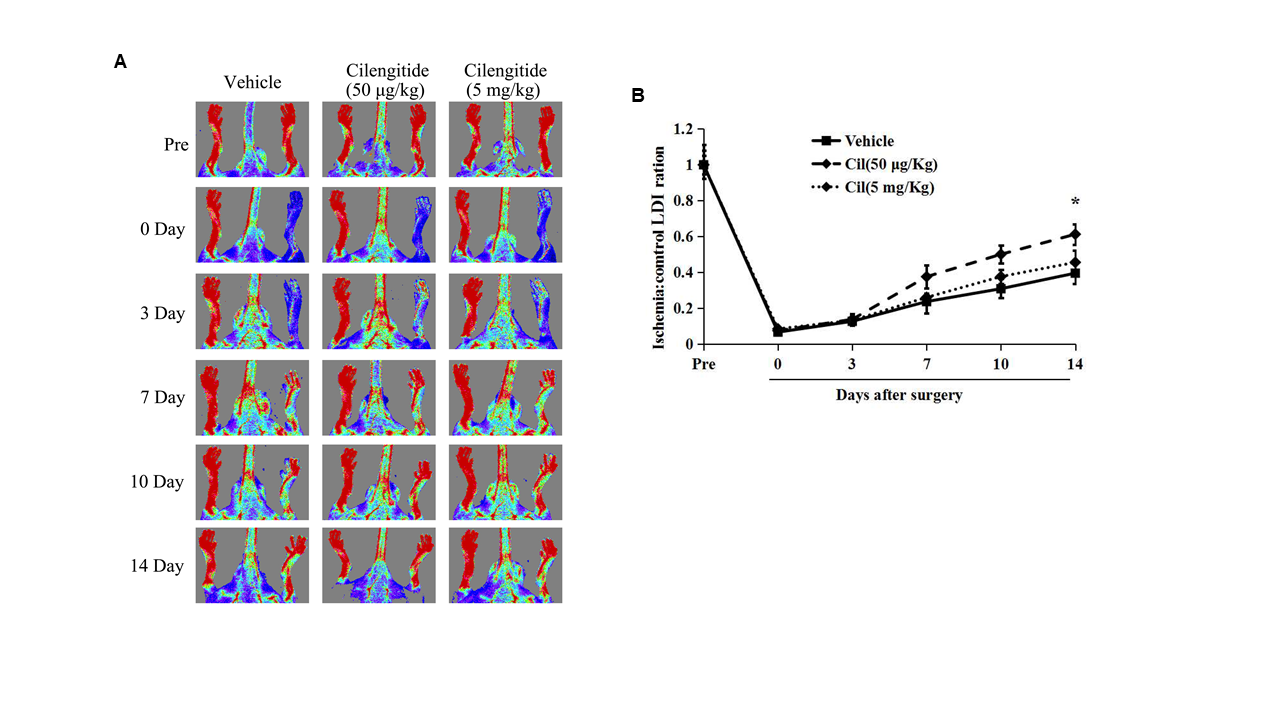

Supplement: Supplementary file 1 [file image1.tif]
